# Colorimetric Paper-Based Sensors against Cancer Biomarkers

**DOI:** 10.3390/s22093221

**Published:** 2022-04-22

**Authors:** Mariana C. C. G. Carneiro, Ligia R. Rodrigues, Felismina T. C. Moreira, Maria Goreti F. Sales

**Affiliations:** 1 BioMark@ISEP, School of Engineering, Polytechnic Institute, 4249-015 Porto, Portugal; mccgc@isep.ipp.pt; 2 Centre of Biological Engineering, Minho University (CEB), 4710-057 Braga, Portugal; lrmr@deb.uminho.pt (L.R.R.); goreti.sales@eq.uc.pt (M.G.F.S.); 3 LABBELS—Associate Laboratory, 4710-057 Braga, Portugal; 4 BioMark@UC, Faculty of Sciences and Technology, Coimbra University, 3030-790 Coimbra, Portugal

**Keywords:** paper-based devices, colorimetric detection, cancer diagnosis

## Abstract

Cancer is a major cause of mortality and morbidity worldwide. Detection and quantification of cancer biomarkers plays a critical role in cancer early diagnosis, screening, and treatment. Clinicians, particularly in developing countries, deal with high costs and limited resources for diagnostic systems. Using low-cost substrates to develop sensor devices could be very helpful. The interest in paper-based sensors with colorimetric detection increased exponentially in the last decade as they meet the criteria for point-of-care (PoC) devices. Cellulose and different nanomaterials have been used as substrate and colorimetric probes, respectively, for these types of devices in their different designs as spot tests, lateral-flow assays, dipsticks, and microfluidic paper-based devices (μPADs), offering low-cost and disposable devices. However, the main challenge with these devices is their low sensitivity and lack of efficiency in performing quantitative measurements. This review includes an overview of the use of paper for the development of sensing devices focusing on colorimetric detection and their application to cancer biomarkers. We highlight recent works reporting the use of paper in the development of colorimetric sensors for cancer biomarkers, such as proteins, nucleic acids, and others. Finally, we discuss the main advantages of these types of devices and highlight their major pitfalls.

## 1. Introduction

**Figure 1:**
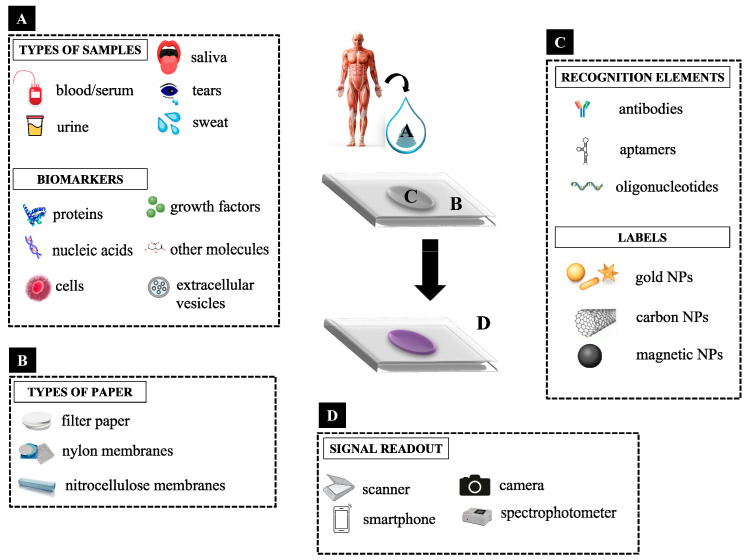
Examples of: (**A**) types of samples and biomarkers, (**B**) types of paper, (**C**) recognition elements and labels, and (**D**) signal readouts used in colorimetric paper sensors.

Cancer is a multifactorial disease [1], caused by mutations or environmental factors [2], and it is one of the major causes of mortality and morbidity worldwide [3]. In cancer, there is an uncontrolled proliferation of cells that grow into an abnormal cell mass called tumor. The primary tumor continues to grow by forming new vessels and metastases spreading to other parts of the body [2]. Cancer diagnosis and treatment is a global challenge [2]. A cancer diagnosis can be performed through image methodologies such as mammography, magnetic resonance imaging, computing tomography, X-rays, ultrasound imaging mass spectroscopy, or biomarkers detection [2]. Biomarkers are molecules that are differentially expressed in normal and disease stages and represent a very large group of molecules as genes, nucleic acid sequences (DNA, RNA microRNA), proteins/enzymes, lipids, small molecules as secondary metabolites, extracellular vesicles or circulating tumor cells (Figure 1A). These molecules are responsible for various functions in our organism, such as storage and transmission of genetic information, catalytic activities, regulation of biological activities, or transport of various molecules, and may be present in tissues such as tumor tissue or in body fluids such as blood, urine, or oral fluid [1,2,3,4]. Several biomarkers have been identified and characterized, being currently in clinical use.

Biomarkers detection could be performed by conventional methodologies that require sophisticated equipment, such as immunoassays, including enzyme-linked immunosorbent assay (ELISA) and polymerase chain reaction (PCR), or by simple paper-based analytical devices (PADs). Immunoassays and PCR are the gold standard in clinical diagnosis for biomarkers detection because they are very sensitive and specific [4,5,6,7,8,9]. Biomarkers detection is crucial for both early diagnosis and prognosis of diseases such as cancer and subsequent timely and suitable treatments to increase survival rates and improve the quality of patients’ life. At least, they can be useful to monitor the patients’ response to therapy and disease recurrence, which is important information in cancer management [1,2,4,5,9,10,11]. For example, cancer patients early diagnosed can have a survival rate of up to 93% [5]. However, biomarkers detection may be difficult because some of them are present at very low concentrations, which commonly occur at the early stages of cancer development [4,12]. Additionally, some biomarkers are related to only one type of cancer, while others are associated with multiple cancers. For these reasons, the detection of a biomarker in complex samples can be challenging and the detection of a single cancer biomarker may not be informative about the disease, hence a panel of biomarkers is often required to guarantee an accurate diagnosis [1,2,4,12]. In addition, according to the organ affected, cancer could be classified into several sub-groups and a specific biomarker can show different levels in different types of cancer [1].

However, these clinical tests require complex and costly protocols with multiple analytical steps, such as washing steps and incubations, high consumption of reagents and samples, and are not available in some regions, especially in developing countries, due to lack of economic resources or trained personnel, which affects accurate diagnosis and further treatment [3,4,5,6,7,8,9,11]. For these reasons, these two procedures (immunoassays and PCR) are not practical in such countries and in places with low resources settings [7,13].

Biosensors are analytical devices composed of a recognition element, also known as a bioreceptor, (e.g., enzymes, antigens or antibodies (Abs), cells, nucleic acids), that recognizes the target and a transducer, (e.g., optical, electrochemical, heat or mass-based) that converts the recognition event into a measurable signal [1]. Biosensors are capable of detecting a single biomarker or a set of biomarkers with high specificity, even when present at low concentrations. For that reason, researchers have focused on the development of biosensor-based techniques to detect various diseases such as cancer, once they can be developed in a point-of-care (PoC) format and be performed outside the laboratory [1,3,9,11]. Although PoC devices are not intended to be substitutes for clinical tests, they have raised an increased interest in the last decade, especially for diagnostic purposes as they could provide a simple and rapid preliminary screening [11,14]. Several diagnostic tests for cancer biomarkers are currently available on the market providing rapid and non-invasive detection [1].

Paper is a versatile material that has been gaining attention as a substrate for the development of PADs in PoC format, being an alternative to the detection of the conventional biomarkers [3,5,14].

Optical and electrochemical transduction techniques are commonly combined with PADs for the detection of several analytes [7,8]. Among optical detection, colorimetry (CM) is a suitable transduction technique for PADs, offering several advantages as a cost-effective, simple, and rapid detection by the naked eye [6,14,15,16,17]. Additionally, the use of smartphones, operative systems, and wireless enable real-time analysis [18,19].

## 2. Paper-Based Sensors

Cellulose is a linear polymer composed of ᴅ-glucose units linked by β-(1,4) glycosidic bonds [12]. It is the most renewable polymer, produced by trees, plants, and some non-pathogenic bacteria [12] and it is available inexpensively in all parts of the world [4]. It is also a weightless, biodegradable, and environmentally friendly material [6,20], that can be easily disposed of by incineration [14]. Moreover, cellulose paper is easy to process, store and transport [4,16]. This polymer exhibits unique physical and physiochemical properties such as biocompatibility, good thermal stability, strength and high sorption ability, hydrophobicity, and high porosity [12]. Paper, due to its low cost and high availability, is an interesting and widely used material for writing, printing, and packaging, but also an excellent platform for chemical and biological analyses [20]. Due to its large number of pores, cellulose paper allows the immobilization of reagents and subsequent drying, which makes it suitable for the development of several devices [5,11,12]. Due to hydrophilic nature of paper and capillary pressure induced by liquids in cellulose fibers, there is no need of pumps for fluid transport, making PADs as lateral flow assays (LFA) and spot tests, power-free [5,11,14,21].

PADs are especially useful for locals with limited resources, as they fulfill the requirements of ASSURED (i.e., Affordable, Sensitive, Specific, User-friendly, Rapid and Robust and Deliverable) concept by WHO (World Health Organization), making them excellent PoC devices [4]. Additionally, they can be combined with portable readers, such as smartphones, making the readout an easy process [22]. PADs are affordable due to the use of inexpensive materials (e.g., cellulose or plastic supports). Nanoparticles (NPs) made from different materials have been used in PADs to meet high sensitivity. In addition, these particles can be easily functionalized with biorecognition elements to assure specificity. PADs are user-friendly once do not require invasive specimens and provide results that are simple to understand, do not requiring trained personnel or sophisticated equipment. They provide a rapid response due to cellulose properties as porosity and capillary forces that accelerates the assay. Constituents of PADs make them robust devices. Finally, the deliverability of these devices enables patients to self-test at home without the need to go to the hospital [3,4,5,7,14,20].

The first paper device was developed in 1956 for semi-quantitative analysis of glucose on urine samples [23] and since then, paper has been used for several application areas as fundamental parameters detection (e.g., temperature, humidity, pH), diagnosis, and therapeutics (urine analysis, immunoassays, drug abuse), food and water quality control and environmental monitoring (e.g., pathogens, pesticides), and forensics (drugs, explosives) [4,14].

## 3. Technical Approaches

Several types of paper have been used in the development of PADs, and should be carefully selected depending on the fabrication method and application of the sensor [6] (Figure 1B). The most used paper is Whatman^®^ paper No. 1 (Maidstone, UK) which has a high absorbency ability, 180 μM of thickness, and a pore size of 11 μM. Other versions of this paper can be used, such as Whatman^®^ paper No. 4 with a large pore size and thus a high retention rate. Filter paper is also being used, with uniform thickness and wicking properties. Nitrocellulose (NC) membranes have smooth surfaces with a pore size of 0.45 μM and are widely used for LFAs, having several chemical functional groups that enable covalent modification with biomolecules and a high degree of retention [6,7]. Bioactive paper, a type of paper that is modified with biomolecules, nylon membranes, and common paper such as conventional printing paper and paper towels is also used to develop PADs [6,17]. Bacterial cellulose nanopaper has also been used due to its optical transparency and flexibility [12].

Despite its inherent properties, cellulose paper can be modified in order to achieve characteristics of interest. Cellulose paper can be modified during its production, by mixing with organic polymers. Otherwise, it can be modified after it is manufactured, by soaking the paper, which enables it to change its surface properties, such as its hydrophobicity [12].

Given the hydrophilic nature of paper and in order to apply it on sensor devices, hydrophobic barriers are often required to restrict the fluid flow of samples and reagents to specific pathways thus preventing their mixing and contamination. Photolithography, inkjet printing, wax printing, screen-printing, chemical vapor-phase deposition, plasma treatment, and laser treatment among other chemical and physical techniques have been used to create these hydrophobic barriers [4,6,24,25].

**Figure 2:**
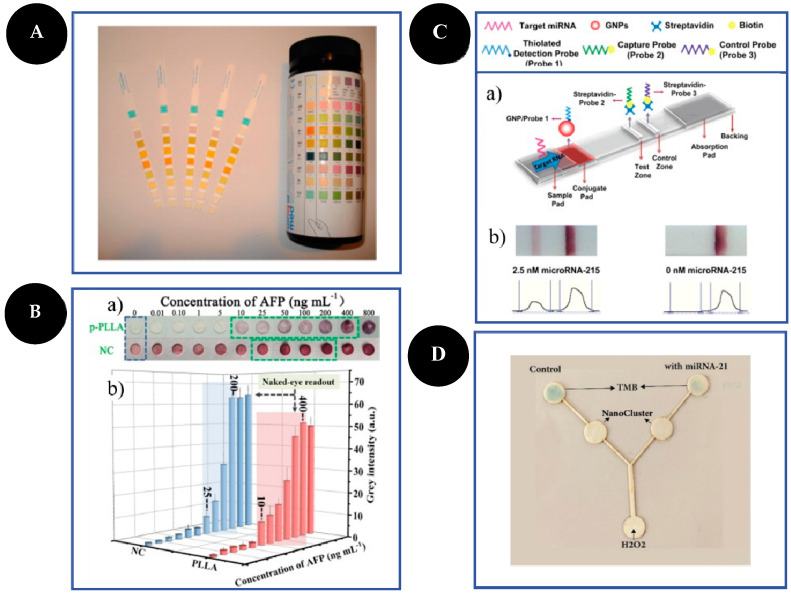
Examples of different types of PADs. Dipsticks (**A**): urine test strips. Reproduced and adapted with permission from [13]; Spot test (**B**): photographs (**a**) and bar charts (**b**) of the corresponding grey values of the colorimetric readout based on a highly porous poly(L-lactic) acid nanofiber and NC platforms for AFP detection. Reproduced and adapted with permission from [30]; LFA (**C**): schematic representation of LFA principle for the detection of microRNA-215 (**a**) and photographs and corresponding optical measurements (**b**) of the developed LFA in the presence (2.5 nM) and absence (0 nM) of microRNA-215. Reproduced and adapted with permission from [32]; µPAD (**D**): photograph of the microfluidic sensor for detection of 1000 pM of microRNA-21 based on peroxidase mimetic activity of DNA-templated Ag/Pt nanoclusters. Reproduced and adapted with permission from [26].

Different types of PADs have been developed and used for analyte detection in different areas such as clinical diagnostics, environmental monitoring, and food quality [5,6,14,26]. Types of PADs include dipsticks (Figure 2), spot tests (Figure 2B), LFAs (Figure 2C), and microfluidic paper-based analytical devices (μPADs) (Figure 2D) [5,8,16]. In this review, which is based on the last 12 years of publications, no dipstick test was found for cancer diagnosis, but several spot tests [18,19,24,27,28,29,30], LFAs [31,32,33,34,35,36,37], and μPADs [22,26,38,39,40,41,42] have been described for cancer biomarkers detection.

### 3.1. Dipsticks

A dipstick is a simple format of a PAD in which a strip of paper is generally impregnated with reagents that change color in the presence of the analyte to give a qualitative response [13]. The first dipstick was developed in 1956 to quantify glucose in urine [43]. Dipsticks are very simple to produce, and their results are very easy to analyze, being directly observed by the naked eye, but it suffers from some drawback as low accuracy and long analysis time, only providing a qualitative response [6,16]. Examples of dipsticks are pH strips or urine test strips that are used nowadays to simultaneously screen multiple disorders as diabetes and kidney disease [8,16,20].

### 3.2. Spot Tests

Spot tests are rapid and inexpensive devices [14] and were applied, for the first time, in the 1930s and 1940s, for metal ion detection using colorimetric ligands [44] and lead to the development of other devices for on-site analysis.

### 3.3. Lateral-Flow Assays

The most known example of an LFA is a pregnancy test [8] but the first LFA was reported and patented in 1956, by Plotz and Singer [45], for rheumatoid arthritis diagnosis. LFAs have evolved over the last decades with efforts to improve their performance [5] and are now a powerful tool for the detection of several biomarkers in clinical contexts but also in environmental monitoring and food safety [8,11].

The principle of an LFA is that a liquid sample, in which the target could or not be present, flows horizontally and without external forces through the several pads of the device, and reacts with previously immobilized reagents [7,46]. LFAs are typically NC strips assembled in a plastic (e.g., polyvinyl chloride) carrier card, containing different parts: sample pad, conjugate pad, flowing membrane or test pad, and adsorbent pad [3,5,7,8,37]. The sample and adsorbent pads are generally made of cellulose paper or glass fibers, while the flowing membrane is usually an NC membrane and the conjugate pad is commonly made of glass fibers [3]. The pads are placed adjacently in order to provide a continuous lateral flow of the solutions once they are added to the sample pad [37]. The sample pad is where the sample should be added and it ensures that the analyte can reach and bind the capture reagents [8,46]. Then, the sample migrates through the conjugate pad which contains colored particles, (e.g., metal NPs), functionalized with recognition elements, (e.g., Abs) responsible for capturing the target [37,46]. The formed conjugate then flows along the strip to the test pad [46]. NC strips modified with different reagents are often used as test pads once they allow the transport of fluids along the strip by capillary forces [5,11]. This pad is the platform for bio-analytical and recognition reactions once it can be easily modified to allow immobilization of capture molecules to form the test and the control line [37]. The presence of the analyte results in a reaction at the test line, while the presence of the color at the control line indicates the adequate flow through the strip [46]. Finally, an adsorbent pad is located at the end of the strip to provide continuous sample flow based on capillary forces and to absorb the excess reagent preventing the backflow of the liquids [8,37,46]. The readout of LFAs consists of the appearance or absence of colored lines on the test line, which can be observed with the naked eye or using a suitable reader [46].

There are two basic assay formats for LFA: sandwich (or direct) lateral-flow assay (sLFA), and competitive lateral-flow assay (cLFA). sFLA is more useful for high-weight molecular compounds and is composed of two separated recognition steps that enhance the specificity and sensitivity. In this approach, the analyte is captured by a primary Ab immobilized on the conjugate pad forming a complex that will be further captured by a transducer, generally a colorimetric probe, conjugated with a secondary Ab and immobilized at the test line. The accumulation of the target at the test line led to the development of a positive signal that is more intense for higher concentrations of the target on the sample. cLFA is more suitable for small molecules, with single antigenic determinants. In this design, the analyte competes and blocks the test line binding sites of the recognition elements, thus preventing their interaction with the conjugates. Higher the concentration of the target the lesser the intensity of the signal [2,3,5,13]. Regarding the biorecognition element used, LFAs can be divided into two main groups: lateral flow immunoassays (LFIA), the most widely used, in which labeled Abs are used as recognition elements for the analyte of interest, coupled with colorimetric or fluorescent readouts [46], and the aptamer-based LFAs in which aptamers are used for target recognition [8]. Regarding signal transduction, colloidal gold NPs (AuNPs) are commonly used as colorimetric probes in LFAs due to their unique optical properties and ease of functionalization for target recognition [5]. Advantages of LFAs include their low-cost, friendly-user format, high shelf life, high sensitivity and specificity, the use of small sample volumes, possibility of multiplex detection, and a wide range of applications [5,12]. However, some drawbacks need to be overpassed. As their sensitivity and specificity rely on membrane matrix and recognition elements, novel materials have been explored for enhancing their performance [5].

### 3.4. µPADs

The first μPAD appeared in 2007, from Whiteside’s laboratory work and was created by a photolithography technique for the colorimetric detection of glucose in urine [47]. Since then, μPADs have been used in many applications as medical ones but also in environmental monitoring, food safety, and forensic analysis [20,48,49]. Unlike LFAs and spot tests, μPAD, flow sample, through the creation of flow channels, which opened the door for multiplex detection in this type of device [11]. These channels are made by using hydrophobic material and applying a pattern onto hydrophilic paper thus defining the reaction zone by the creation of hydrophilic channels [7]. On two-dimensional (2D) μPADs, channels are created by chemical or physical hydrophobic barriers, through several techniques such as photolithography, wax printing, screen-printing, inkjet printing, and plasma oxidation, whereas three-dimensional (3D) μPADs are constructed by folding several layers of patterned paper [7,8,11,22,48,49]. The choice of the fabrication technique should be done considering the cost, the substrate used, the fabrication time and the equipment availability. Whatman^®^ (Maidstone, UK) filter paper No1 is generally used in this type of devices due to uniform thickness and wicking properties. μPADs only require very small amounts of fluids (5 to 10 μL) and provide faster responses than the current laboratory techniques. In addition to that, μPAD offers the advantage of miniaturization, disposability, multiplex analysis by the creation of several channels, and simultaneously semi-quantitative and quantitative responses [7,48,50]. It also allows on-site analysis which is an advantage for developing countries [49]. Despite all the advantages mentioned above and many efforts (including the use of pre-concentration techniques), μPADs can suffer from a lack of sensitivity.

## 4. Receptors or Biorecognition Elements

The selection of recognition elements depends on the target we intend to detect. Abs and aptamers are the most common receptors used in the development of PADs for cancer biomarker detection. In the case of nucleic acids as targets, oligonucleotides and aptamers are usually applied; on the other hand, for proteins, enzymes, and cells detection, Abs and aptamers can be used [3] (Figure 1C). Despite these well-established recognition elements, molecular imprinting polymers emerged as alternative recognition elements, displaying high selectivity, as well as fast responses and low costs [51].

The immobilization of the receptors should take place both on the paper surface and in the nanomaterials that are used as probes. Receptors could be immobilized on the paper matrix either by physical or chemical methodologies. These strategies should enable the retention of molecules without comprising their bioactivity. For the immobilization of receptors as Abs on tags, adsorption techniques or covalent binding are usually employed. In the case of aptamers, 5′-labelled sequences with thiol or amine groups could be used for attachment to NPs. In addition to that, the streptavidin-biotin interaction is commonly used for their immobilization on the NC membrane [3].

## 5. Signal Transduction

Optical and electrochemical techniques are usually coupled to PADs to detect the analyte of interest as, CM, fluorescence, chemiluminescence (CL), surface plasmon resonance (SPR), surface-enhanced Raman spectroscopy (SERS), electrochemistry, photoelectrochemistry, or electrochemiluminescence [4,6,7,8,9,14]. Optical-based sensors for cancer have been developed making use of the advantages of optical transducers and nanomaterials [2]. Optical detection is based on the measurement of an optical signal that could be absorption, transmittance, fluorescence, and CL generated upon recognition of the analyte [4]. Among these, CM is the most suitable as results can be viewed from the naked-eye [6,15,16]. So, this review will focus on colorimetric detection for PADs.

### 5.1. Colorimetric Transduction

Colorimetric paper-based sensors have emerged in the diagnosis of different diseases including cancer [22], neurodegenerative [52], infectious [53], and other chronic diseases [54].

Colorimetric assays detect the absence or presence and respective concentration of the analyte of interest through the evaluation of color production or color change. This production or change of color could be induced by dyes, enzymes or NPs as AuNPs [6]. Colorimetric assays evaluate absorbance or reflectance intensity changes due to chemical or biochemical reactions between the target and chromogenic substances used as probes. Absorbed or reflected light intensity generally results from optical properties changes due to SPR or structural shifts [4,18]. This is one of the most common detection techniques used in PADs as it enables a simple, cost-effective, real-time, and on-site detection of several molecules [17]. In addition, paper provides a bright background with high contrast over color development [14]. Additionally, samples to be analyzed in colorimetric PADs can be obtained by invasive collection as blood, serum, or synovial fluid, or by the minimally invasive or non-invasive way, as tears, saliva, urine, or sweat [17] (Figure 1A). Despite the reported advantages, sometimes CM suffers from a lack of selectivity and sensitivity originating heterogeneous signals that could be misunderstood by the users [4].

Several types of nanomaterials and nanostructures have been used as carriers, targeting mediators, or detection interfaces on PADs. Paper is modified with these NPs enabling the improvement of several parameters of the sensor as sensitivity, the limit of detection (LOD), range of detection, selectivity, and specificity against interfering species [9,12]. Catalytic reactions or enzymatic conversion of chromogenic substrates is one of the most common strategies used in colorimetric sensing and is possible due to a reaction between an enzyme and its substrate thus forming an enzyme-substrate complex that produces color [6,42]. Enzymes such as peroxidase have been used once they catalyze the oxidation of substrates to produce a color change [27]. However, enzymes are natural molecules and have some inherent disadvantages such as stability, sensibility to environmental conditions, and some drawbacks associated with their production and modification. To overcome this, efforts have been made in order to mimic peroxidase-like activity by producing catalytic nanomaterials [27]. It has been found that some materials such as metal nanoclusters have enzyme-like activity and could catalyze 3,3′,5,5′-tetramethylbenzidine (TMB) in the presence of hydrogen peroxide thus leading to a blue colourful product. This methodology has been used for the detection of cancerous cells [42]. NPs are also used as probes once they are generally easy to synthesize with different shapes and from several materials and are easily functionalized (Figure 1C). These include metal NPs as AuNPs or silver NPs (AgNPs), magnetic NPs (MNPs), paramagnetic particles, cerium oxide NPs, and carbon NPs as multi-walled carbon nanotubes (MWCNTs) and reduced graphene oxide (GOx) that leads to higher surface areas and magnetic particles, which allows concentrating the target at the detection zone [12,27,36]. When label NPs are used, they should be chosen according to several parameters such as colloidal stability, the efficacy of conjugation with recognition elements, and low non-specific binding [46]. Colloidal AuNPs are an attractive nanomaterial for colorimetric biosensing and the most widely used label showing great potential to increase sensitivity in colorimetric biosensing. It occurs due to their intense color in consequence of their unique chemical and physical properties as optical ones and plasmonic fields, ease of preparation with different shapes, from several materials and through well-established and inexpensive synthesis protocols, and high stability booth in solution and dried form [12,27,35,46]. They are used to bind to secondary Abs in immunoassays and their aggregation/disaggregation due to specific interaction with the analyte induces a change of color [6]. AuNPs solutions have a red color due to their localized surface and changes in their surface can change this color from red to purple [35].

### 5.2. Signal Readout

Color changes provided by colorimetric sensors could be either qualitative, observed by comparison of the color by the naked eye, or enable a quantitative result by using readout devices that capture the results as simple instruments such as scanners, cameras, smartphones, or spectrophotometers [3,4,5,6,7,16,17] (Figure 1D). Smartphones are highly used due to their useful properties as easy-of-used, high-resolution cameras and operative systems, and wireless connectivity thus enabling real-time analysis [18,19]. Image capture should occur under controlled light conditions to avoid affecting the sensibility and repeatability of the assay. Black or white boxes with controlled light conditions have been developed for image capture [4,5]. Fluorescent labels or paramagnetic particles cannot be read by the naked eye and need specific readers for quantitative measurements [46]. After capture, images are transferred to a device as a computer where specific software is installed, capable to calculate parameters such as red, green, and blue (RGB) space or hue, saturation, and value (HSV) space. Therefore, the concentration of the target is calculated, enabling its quantification [55]. Real-time analysis and portability are important features of PoC devices and are dependent on the miniaturization and development of portable readout devices [2]. Regarding colorimetric sensors, although the instruments required for image processing are portable, they can be larger and heavier than the PAD itself. For this reason, the naked human eye is the most suitable detector for on-site analysis [55]. However, the naked human eye is very sensitive to many factors such as color intensity, tonality, and light which leads to bias in the user’s interpretation. For this reason, many researchers have developed new display formats that enable accurate visual recognition and miniaturization of the devices, such as distance-based, counting-based, text-based, and time-based readouts [55,56]. Among the different types of PADs, μPADs are the most suitable for miniaturization, especially due to the smaller dimensions of the channels used on microfluidic devices [2].

## 6. Application of Colorimetric Paper-Based Sensors in Cancer Biomarkers

**Figure 3:**
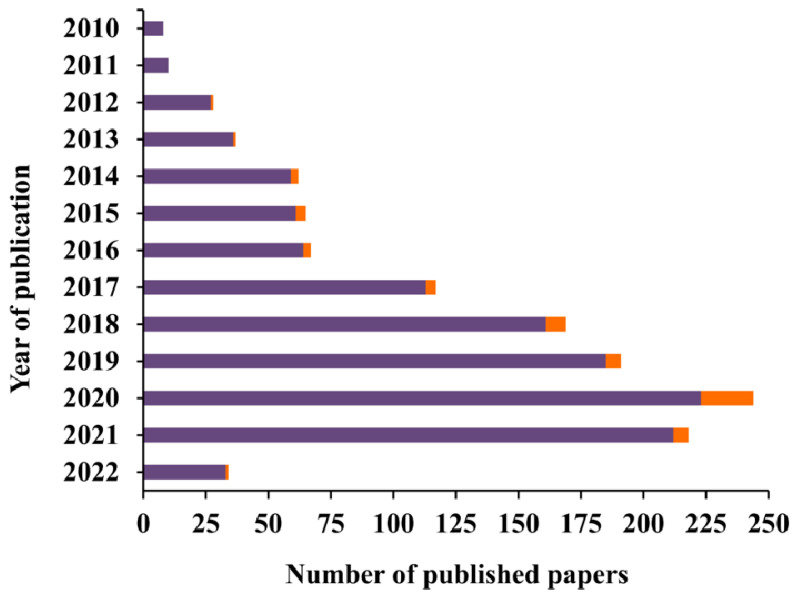
Number of published papers by year of publication. Data obtained from ISI WEB OF KNOWLEDGE with the keywords “colorimetric AND paper-based” (purple bar) and “colorimetric AND paper-based AND cancer” (orange bar). Data collected in March 2022.

In the last decade, the number of reports published on colorimetric paper-based sensors has been increasing (Figure 3), being the ones applied to cancer a minority but still growing over the years.

Several works reporting the use of PADs for cancer biomarkers using optical transduction as chemiluminescence and fluorescence have been published. However, in this review, we will only focus on colorimetric detection. Table 1 summarizes several works reporting colorimetric paper-based sensors for different types of cancer biomarkers, over the last 12 years.

### 6.1. Proteins

Proteins are biomolecules that play an important role in our organism and their abnormal expression can lead to several diseases, such as cancer. Proteins can be produced by cancer cells and released into biological fluids such as blood and urine. The main problem related to the detection of cancer proteins is their low levels compared to other proteins. Enzymes are proteins and changes in their activity are also related to the development of diseases such as cancer [3].

Carcinoembryonic antigen (CEA) is a cell surface glycoprotein, and a well-known biomarker for many cancers including breast, liver, gastric, and colorectal cancers [4,10,15].

Colorimetric detection of CEA for gastric cancer diagnosis was performed by Wang and colleagues [22] based on a sandwich immunoassay and a wax-printed multilayer μPAD. A primary Ab was immobilized onto Whatman^®^ filter paper, and a second Ab coated with horseradish peroxidase (HRP) recognizes the target. Then, a TMB solution was applied as a color indicator, leading to a blue color whose intensity increases proportionally to the target concentration. This work includes a smartphone-based detection system that calculates and displays the analyte concentration on the screen. The current method showed a linear range from 0.5 to 70 ng/mL with a limit LOD of 0.015 ng/mL.

**Figure 4:**
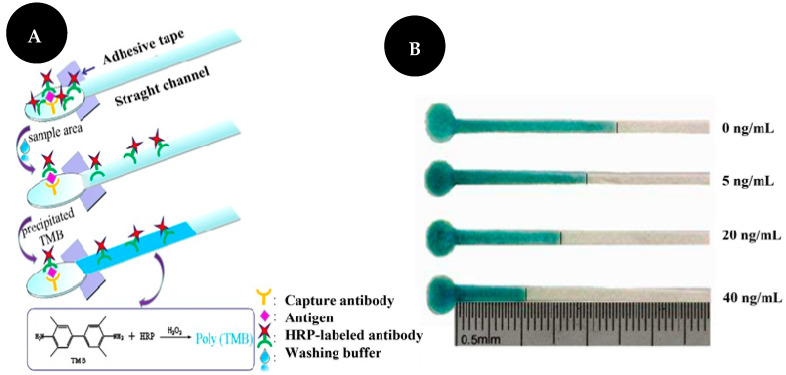
Scheme of distance-based detection of CEA (**A**). Results of CEA detection (**B**). Reproduced and adapted with permission from [15].

Chen and co-workers [15] developed a distance-based immunosensor on a μPAD for the semi-quantitative detection of CEA (Figure 4). Firstly, a capture Ab was immobilized on the sample area after oxygen plasma treatment. Then, the target and an HRP-modified Ab were added. The TMB-hydrogen peroxide system was used as chromogenic and its interaction with HRP-labelled Ab led to the development of a visible blue bar that is inversely correlated to analyte concentrations, from 0 to 40 ng/mL. This work enables CEA detection based on the length of the colored band, which can be observed with the naked eye without the use of external equipment such as a camera or scanner. CEA detection was carried out in serum and levels as low as 5 ng/mL could be detected by this device. The LOD was set at 2 ng/mL.

Alizadeh and co-workers [24] developed a microfluidic paper-based immunosensor for CEA detection. Primary Abs functionalized with chitosan were mixed with an ionic liquid and immobilized on the paper surface through glutaraldehyde (GA) cross-linking method. The function of the ionic liquid was to avoid nonspecific binding. After sample incubation, Co_2_(OH)_2_CO_3_-CeO_2_ nanocomposite functionalized with secondary Abs was added to the sensor. Then, the peroxidase-like activity of the nanocomposite led to TMB oxidation in the presence of hydrogen peroxide and consequently to a change of color. Finally, sulfuric acid is added and the color change from blue to yellow. The observation of the color is possible by the naked eye and quantitative results were achieved by image capture by a smartphone and its further analysis with specific software. The intensity value of the color is proportional to CEA concentration and enables its detection with a linear range from 0.002 to 75 ng/mL and a LOD of 0.51 pg/mL.

A colorimetric immunosensor for CEA detection was prepared by Liu and co-workers [27] based on ZnFe_2_O_4_-multiwalled carbon nanotubes (ZnFe_2_O_4_-MWNTs) peroxidase-like activity. Primary Abs were entrapped on chitosan and porous gold layers deposited onto the paper surface and the ZnFe_2_O_4_-MWNTs were functionalized with secondary Abs by using (3-aminopropyl)triethoxysilane (APTES) and GA as coupling agents. The oxidation of TMB in the presence of hydrogen peroxide catalyzed by the nanocomposite leads to a color change visible by the naked eye and digitalized for a more accurate result. Grey intensity values were obtained by ImageJ and it was shown that these values increased directly with increasing concentrations of CEA. It obtained a linear range from 0.005 to 30 ng/mL and achieved a LOD of 2.6 pg/mL.

A paper-based immunoassay for CEA detection was developed by Nascimento and co-workers [10]. Paper-based microplates were designed by printing wax patterns to create hydrophobic barriers. Abs against CEA were immobilized on the microzones and the sample was incubated. Then, Abs labeled with biotin were bound and finally, an avidin-peroxidase solution and TMB were applied for color development. The reaction was stopped with and acidic solution and the color of the product changed from blue to yellow. The mean pixel intensity was calculated using Adobe Photoshop^®^.

**Figure 5:**
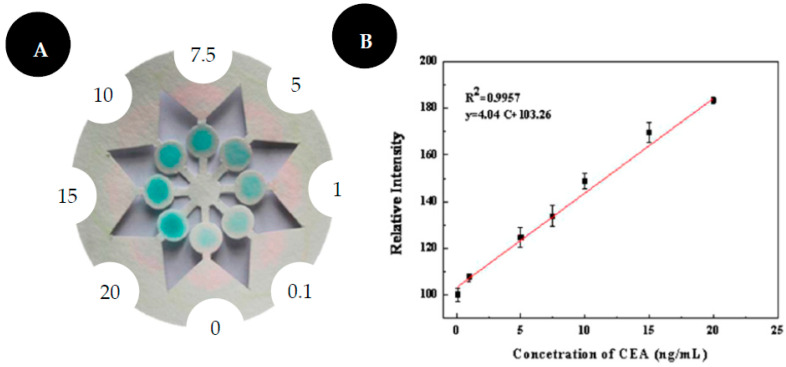
Scheme of ring-over device for CEA detection (**A**). Results of CEA detection (**B**). Reproduced and adapted with permission from [38].

Liu and colleagues [38] developed a colorimetric immune system for CEA detection in human serum, with eight microfluidic channels created by the paper cutting fabrication method (Figure 5). This device integrates a ring-oven technique, in which the nonspecific binding protein can be easily washed away from the detection area to the waste by capillary forces. This washing process includes an oven in which the filter paper is adapted. The washing buffer is dropped on the middle of the paper and the nonspecific binding protein were carried to the border of the paper circle and then its solvent is evaporated from the filter paper due to the existence of the heated ring-oven. The paper was modified by the chitosan coating and GA cross-linking method to enable the covalent immobilization of Abs. Hrp-labeled Abs were used to catalyze TMB in the presence of hydrogen peroxide. A blue color is formed and observed by the naked eye. To obtain quantitative data, the results were captured by a scanner and analyzed with ImageJ. The greyscale was used, and its value is inversely proportional to CEA concentration. This device achieves a LOD of 0.03 ng/mL and a good linearity was obtained from 0.1 to 20.0 ng/mL of CEA.

Cancer antigen 125 (CA-125) is a mucin-like glycoprotein, also known as mucin 16 (MUC-16), used to monitor epithelial ovarian cancer [18,19].

Hosu and colleagues [19] developed a colorimetric paper-based immunosensor for CA-125. In this work, a sandwich strategy was proposed, in which a primary Ab was immobilized onto an NC membrane to detect CA-125 that is further sandwiched by AuNPs labeled with a secondary Ab. A silver enhancement solution is employed, and the AuNPs-Ab complex induces silver deposition leading to the formation of gold-silver NPs that produce different grey color spots dependent on CA-125 concentration. A smartphone camera coupled to a homemade carton dark box was used as a color detector, performing the image capture and data processing. A LOD of 30 U/mL was achieved in a linear range from 30 to 100 U/mL.

Alpha-fetoprotein (α-AFP) is a known marker of many diseases such as antenatal screening or several cancers, such as liver cancer [18].

Aydindogan and co-workers [18] work describes an immunosensor for CA-125 and α-AFP detection, two well-known biomarkers for liver and ovarian cancer, respectively. Cysteamine conjugated AuNPs (Cys-AuNPs) were firstly immobilized on an NC membrane and then Abs (anti-AFP and anti-CA-125) were conjugated via GA cross-linking to the NP on the detection pad. Different concentrations of α-AFP and CA-125 were applied to the spots on the NC membrane and the visual colorimetric changes due to optical properties of AuNPs were captured by a smartphone and color was analyzed by ImageJ. The linear range for α-AFP was found to be from 0.1 to 100 ng/mL with a LOD of 1.054 ng/mL. For CA-125, the linear range was 0.1 to 10 ng/mL and a LOD of 0.413 ng/mL has been achieved.

A microfluidic colorimetric paper-based immunodevice for multiplex detection of α-AFP and CEA was developed by Liang and colleagues [41]. Palladium (Pd) NPs/Fe_3_O_4_@C were produced by in situ growth of Pd NPs on Fe_3_O_4_@C MNPs. A sandwich-based approach was constructed by entrapping capture Abs on flower-like AuNPs (FLAuNPs) onto the chitosan-modified paper surface. Pd/Fe_3_O_4_@C NPs functionalized with secondary Abs were used to mimic peroxidase and thus catalyze reactions of the chromogenic substrates, TMB and orto-phenylenediamine (OPD). A fast color change was visible by the naked eye, being a blue color obtained in the presence of CEA due to TMB reaction, whereas an orange color was obtained in the presence of α-AFP due to OPD reaction. The results showed a good colorimetric response for grey intensity values between 0.005 and 30 ng/mL and a LOD of 1.7 pg/mL was achieved for CEA and α-AFP.

A highly porous poly(L-lactic) acid nanofiber membrane (p-PLLA) was developed by Ma and co-workers [30] for sensitive and selective detection of α-AFP. p-PLLA was fabricated by electrospinning technique and immobilized with the capture Ab. The addition of the target molecule followed by AuNP-labeled Abs enabled the formation of a sandwich immunocomplex allowing a naked-eye detection through a change of color from colorless to red. The red color becomes deeper with the increase in target concentration from 10 to 400 ng/mL, with a LOD of 0.17 pg/mL.

Prostate-specific antigens (PSA) have been used for diagnosis, screening, and postsurgical monitoring of prostate cancer [25].

Nie and co-workers [25] proposed a simple method for the preparation of μPADs and applied it to a colorimetric immunoassay coupled with gold enhancement amplification for PSA detection, as a model target. Permanent markers were used to plot designed patterns on the paper surface by using metal templates with no need for specific equipment and trained personnel. That marks will act as hydrophobic barriers and define hydrophilic flow paths. A primary Ab was immobilized on a paper surface to recognize the target. After target incubation, AuNPs modified with a secondary Ab were added and finally, a gold enhancing solution was applied. PSA concentrations were detected in a range from 0.5 to 50 μg/L and the LOD was determined to be ≈360.2 ng/L.

A photothermally responsive poly (methyl methacrylate) (PMMA)/paper hybrid disk (PT-disk) was developed by Fu and co-workers [57] for PSA detection on a paper substrate. This work is based on a sandwich immunoreaction with colorimetric detection due to the conversion of iron oxide to Prussian blue NPs (PBNP). Aldehyde-modified cellulose paper was incubated with anti-PSA Abs for PSA recognition. Fe_3_O_4_ NPs labeled with anti-PSA were added to form an immunocomplex and a color change from brown to blue is observed as a result of Fe_3_O_4_ to Prussian blue conversion. An increase in color intensity was linearly correlated with PSA concentration in a range from 3 to 80 ng/mL with a LOD of 2.7 ng/mL.

Prostate cancer antigen 3 (PCA3) is a tumor marker, is overexpressed in prostate cancer [58]. An on-chip reverse transcription loop-mediated isothermal amplification (RT-LAMP) for colorimetric detection of PCA3 was developed by Wang and colleagues [58]. This device is composed of three functional units: a 3D polymer/paper hybrid chip, a thermal unit, and an imaging box. Reagents and RNA samples were loaded on the amplification pad, which is a sponge-like polyvinyl alcohol (PVA) pad, placed on the thermal module plate. After amplification, a stick was pressed onto the amplification pad and RT-LAMP product was introduced to the colorimetric detection zone, which is a chromatography dry paper containing a chromogenic substrate. Naked-eye colorimetric changes were observed and a LOD of 0.34 fg/μL RNA was achieved.

p16^INK4a^ is a cyclin-dependent kinase inhibitor, being a biomarker of cervical cancer once it is overexpressed in human papilloma virus (HPV)-associated precancerous cells [28].

A paper-based immunosensor was developed by Yokchom and co-workers [28] for p16^INK4a^ detection in cervical cancer, with a visual readout of 30 min. AuNPs functionalized with anti-p16^INK4a^ were used as probes and immobilized on the NC membrane. The signal amplification strategy was based on the combination of the peroxidase activity of HRP-Ab conjugate and the peroxidase-like activity of AuNPs. In the presence of an immune complex between the target and the Ab on the reaction pad, HRP catalyzes the oxidation of TMB, producing a visible naked-eye blue-colored spot, whereas in the absence of the target, a red coloration prevenient from AuNPs is observed. The results were captured with a mobile phone camera and analyzed by ImageJ. Four cervical cancer cell lines were analyzed and the lowest number of cells that could provide a positive visible signal was defined as the LOD. HeLa, CasKi, and SiHa cell lines displayed a clear positive signal, with a LOD of 300, 300, and 3000 cells.

Additionally, for cervical cancer, HPV 16/18 E6 oncoprotein has been identified as a potential biomarker, especially useful in prognostic. It has the ability to detect pre-cancer stages thus providing an earlier diagnosis [29].

HPV 16/18 E6 oncoprotein was detected by Mwai and colleagues [29] in a paper-based dot blot approach. HRP labeled Abs for HPV were conjugated with AuNPs and used as probes for the oxidation of TMB. The use of AuNPs enabled a signal enhancement of peroxidase activity thus providing an assay with higher sensitivity when compared with non-conjugated Abs. A color change from colorless to blue and the LOD was 0.0005 pg/mL.

Valosin-containing protein (VCP) is a potential biomarker for the early detection of cervical cancer, is related to HPV transformation [59].

**Figure 6:**
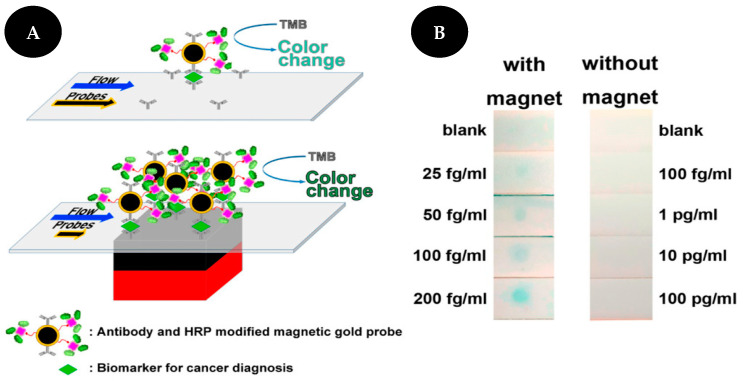
Scheme of VCP detection (**A**) without (**upper**) and with (**bottom**) magnet. Comparison of detection results of VCP (**B**) with (**left**) and without (**right**) magnet. Reproduced and adapted with permission from [36].

Ren and collaborators [36] proposed a magnetically focused lateral flow sensor (mLFS) for VCP detection, applied to cervical cancer, using Fe_3_O_4_ core/gold shell nanostructures labeled with Abs for target recognition and an enzyme for signal amplification (Figure 6). An external magnet is placed under the strip so that the MNPs control the movement of the target in the strip and consequently increase the contact time of the target labeled with gold-coated magnetic probes at the detection zone. Additionally, an enzyme-based amplification strategy is used to increase the sensitivity of the assay allowing a naked-eye detection of as low as 25 fg/mL of VCP in PBS and 16 fg/mL of VCP in a mixture of proteins extracted from tissue lysate samples.

Sarcosine is an amino acid known as a potential indicator of prostate cancer as it is present in low levels in healthy individuals but at high concentrations in prostate cancer patients, being a non-invasive biomarker that can be found in urine samples [60,61].

Nascimento and colleagues [60] presented a microfluidic enzymatic device that can be used for preliminary and non-invasive screening of sarcosine in urine samples. A paper-based microzone plate was printed by wax printing technique on a Whatman^®^ paper filter. First, sarcosine oxidase was added to each spot followed by the addition of HRP. When sarcosine is present in the sample, sarcosine oxidase catalyzes sarcosine demethylation, producing hydrogen peroxide. Subsequently, HRP will catalyze the reduction of hydrogen peroxide, oxidizing the 2,2-azino-bis(3-ethylbenzothiazoline-6-sulfonic acid) diammonium salt (ABTS) redox indicator. The oxidized ABTS leads to the formation of a blue-green coloration. The results were digitalized, and the average intensity of the color was obtained by Adobe Photoshop^®^ CS4, showing that a linear range up to 1 mM was obtained, with a LOD of 0.21 mM.

Masumoto and colleagues [61] developed a paper-based colorimetric assay for sarcosine. Whatman^®^ filter paper no. 1 was patterned to form hydrophobic barriers by wax printing methodology. In this work, sarcosine oxidase (SOx) selectively oxidizes sarcosine present in the sample thus producing hydrogen peroxide that will be detected through the reaction of TMB and HRP, resulting in a blue-colored product. A signal-enhancement technique was applied by the modification of the paper with a cationic polymer, poly(allylamine hydrochloride) (PAH), resulting in an improvement of sensor response. This modification provided a physical barrier that limits the enzymatic reaction to the top surface of the substrate and prevents the washing-out effect of enzymes and colorimetric products, with subsequent stronger color development and more uniform coloration. Red color intensity obtained by ImageJ showed a linear detection of sarcosine between 0 and 10 μM. The presented modification with PAH enhanced the sensitivity of the sensor, lowering its LOD from 12.6 μM (unmodified substrate) to 0.6 μM and also improving the assay precision. Assays in artificial urine showed some interference for samples with higher pH. This effect was overpassed by lowering sample pH and a LOD of 2.5 μM was achieved.

Cytochrome C (Cyt c) measurement has been used for the early detection of various cancers [39].

Mesgari and colleagues [39] developed a colorimetric µPAD aptasensor for sensitive detection of Cyt c, using β-Co(OH)_2_ nanoplates anchored to mesoporous carbon as a catalyst for TMB oxidation and specific aptamers for Cyt c as recognition elements. The presence of Cyt c will promote the detachment of aptamers from nanoplates, thus exposing its peroxidase-like activity that causes the oxidation of TMB. A naked-eye visible color change on the Whatman^®^ paper occurs, with an increase in blue color due to increasing concentrations of Cyc c. The reported aptasensor has a wide linear range from 1 µM to 1 mM with a LOD of 5.0 × 10^−7^ M for colorimetric detection.

Interleukin-6 (IL-6) is a cytokine involved in many biological processes, playing an important role in the development of several illnesses such as inflammatory ones like cancer, in which its levels are increased [31].

**Figure 7:**
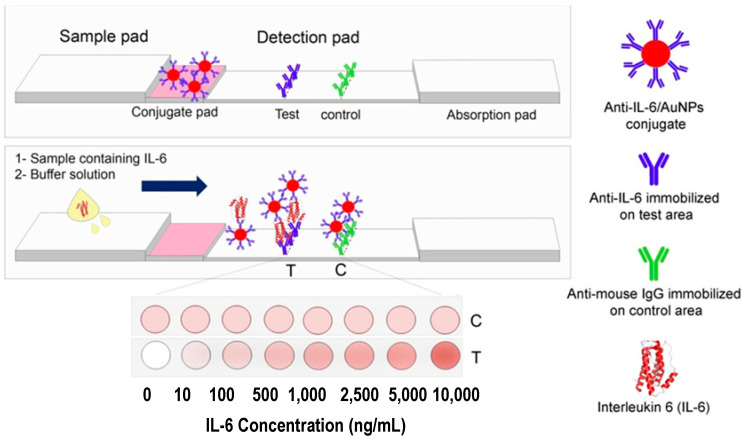
Scheme of IL-6 detection by LFIA. Reproduced with permission from [31].

Sene and co-workers [31] developed a colorimetric LFIA based on a sandwich approach for IL-6 (Figure 7). Abs against IL-6 were conjugated with AuNPs and immobilized on a conjugate pad. When IL-6 is present in the sample, it binds to the previously mentioned conjugate. Therefore, the complex formed between IL-6 and AuNPs-Ab conjugate will bind with Abs immobilized on the test pad thus producing a naked-eye visible red color whose intensity is proportional to IL-6 concentration. For quantitative measurements, the images were captured, and the color intensity was obtained by image analysis in ImageJ. This assay enables rapid (20 min) IL-6 detection in a linear range from 1.25 to 9000 ng/mL with a LOD of 0.38 ng/mL.

### 6.2. Enzymes

Telomerase is a well-known cancer biomarker once its overexpression has been identified in several solid tumors [62].

Mahmoudi and colleagues [62] developed a colorimetric paper-based platform for fast and equipment-free naked-eye detection of telomerase activity due to a color change in response to enzyme activity. In this assay, hybridization occurs between a telomere complementary oligonucleotide coupled to AuNPs and telomerase elongated biotinylated probe. Avidin-biotin interaction enables the attachment of the assembly and then the enlargement of the AuNPs by the hydroxylamine hydrochloride strategy enables a signal amplification leading to a naked-eye color. A smartphone was used for image capturing and ImageJ was employed for data handling. The analytical performance was evaluated with the enzyme extracted from breast cancer cells and visual detection of telomerase activity could be achieved down to 6 cells, with a detection range from 6 to 25,000 cells. The paper strip color changed from light-red to light-red-blue and finally to black-red with increasing telomerase concentration.

**Figure 8:**
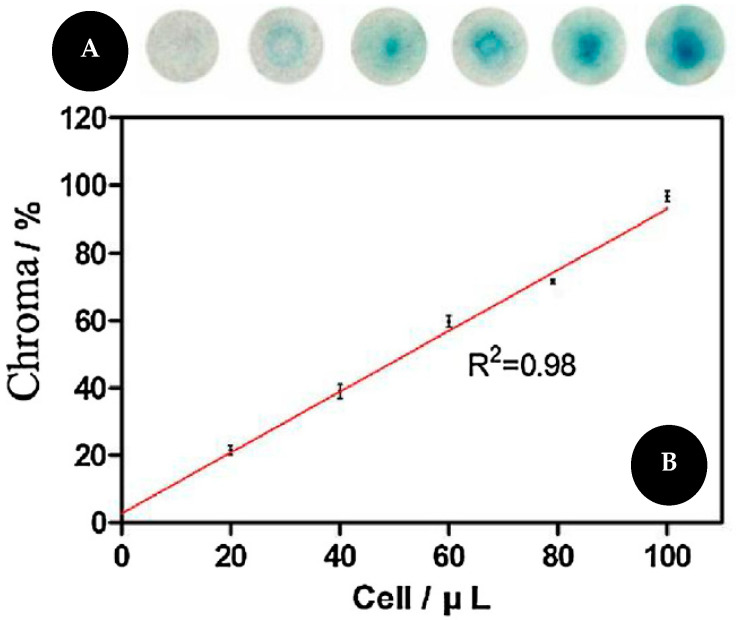
(**A**) Pictures of the colorimetric assay for telomerase activity (**B**) and corresponding graphic with linear relationship between MB and telomerase concentration. Reproduced and adapted with permission from [63].

Fu and co-workers [63], presented a colorimetric assay for telomerase activity detection based on telomeric elongation and capturing amplification using methylene blue (MB) as a colorimetric probe on functionalized cellulose paper (Figure 8). The telomerase substrate (TS) was dropped on sterile cellulose paper. This primer will be extended by telomerase and produce a long single DNA (sDNA) that will further capture more probes thus increasing the sensitivity of the assay. Oligonucleotides labeled with MB will hybridize with sDNA leading to a color change. Signal intensity is associated with the quantity of sDNA and thus with telomerase activity. When the samples are absent of telomerase, oligonucleotides cannot hybridize with sDNA. For quantitative results, the images were captured and analyzed by Adobe Photoshop^®^ CC and it showed an LOD of 20 cells/μL.

Alkaline phosphatase (ALP) is a hydrolase enzyme that catalyzes the hydrolysis of monoesters of phosphoric acid. It constitutes a biomarker for the diagnosis of several illnesses, including breast and prostate cancers [40].

Stanlei and co-workers [40] designed a colorimetric paper-based PoC device for ALP detection. The paper was patterned by a commercial wax printer. Para-nitrophenylphosphate (PNPP) was used as a colorimetric probe and immobilized in the reagent zone. Then, ALP was dropped on the reagent zone, and it changes to yellow color in the presence of ALP, due to dephosphorilation reaction. The intensity of the color is proportional to the target concentration and the results were captured with a digital camera for RGB and HSB calculation using MATLAB software to extrapolate the target concentration. It was shown a linear relationship between 100 and 500 U/L for red intensity values and a linear range between 30–500 U/mL for saturation values.

Pseudopodium-enriched atypical kinase one, SGK269 (PEAK1) was identified as a potential biomarker for pancreatic ductal adenocarcinoma (PDAC) [64].

Prasad and collaborators [64] developed a paper-based colorimetric immunosensor to detect PEAK1 in an hour. Capture Abs for PEAK1 were immobilized onto chromatographic paper for PEAK1 recognition. AuNPs functionalized with anti-PEAK1 Abs were used as probes to establish an immunocomplex with PEAK1. Finally, signal amplification of the colorimetric reaction occurred by the addition of hydroxyl naphthol blue (HNB) whose degradation is catalyzed by the AuNPs in the presence of sodium borohydride. A naked-eye color change from blue to colorless can be observed in the presence of PEAK1 and ImageJ was used to measure grey values whose intensity linearly increased with the increase in PEAK1 concentrations from 1 × 10^−9^ g/mL to 1 × 10^−5^ g/mL. A LOD of 1 ng/mL was achieved and it was 10-fold lower than that obtained without the use of HNB for signal enhancement.

### 6.3. Growth Factors

Epidermal growth factor receptor 2 (HER2) is a biomarker for early cancer diagnosis once it is known to be overexpressed in many types of cancer such as breast, lung, gastric, ovarian, and oral cancer. It was shown its upregulation in 20 to 30% of aggressive breast cancer cases showing high concentrations in the blood of these patients [35].

**Figure 9:**
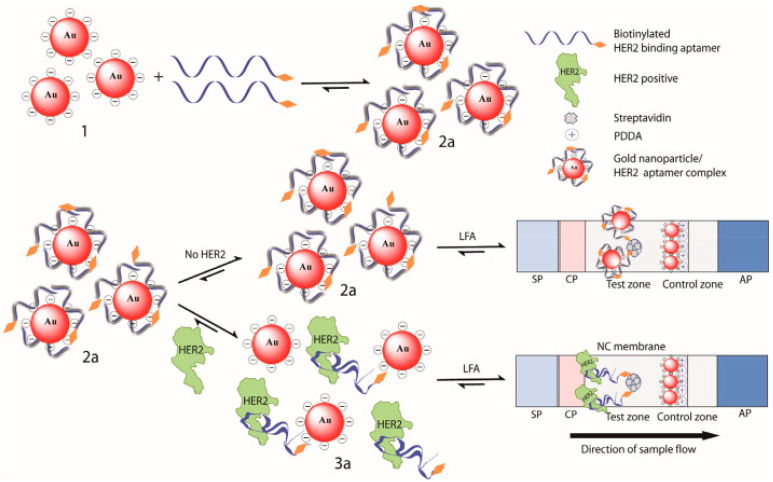
Scheme of HER2 detection by an adsorption-desorption colorimetric LFA. Reproduced with permission from [35].

A colorimetric LFIA using an aptamer as a biorecognition element was developed by Ranganathan and colleagues [35] for HER2, using AuNPs as colorimetric probes (Figure 9). First, biotin functionalized aptamers were attached to AuNPs, preventing their aggregation, and leading to a red color solution. When HER2 is present in the sample, it bonds to aptamers, leading to the release of AuNPs. The solutions were then applied in an LFA format. In the absence of the target, the AuNP-aptamer bounds to streptavidin attached to the membrane and leads to a red dot in the test line. When the target, HER2, is present, aptamers bind to it forming a complex. This complex will further be captured by streptavidin, immobilized on NC. Once there are no AuNPs on the complex, the red dot does not appear on the test line. This assay showed a LOD of 20 nM and 24 nM in water and human serum, respectively.

### 6.4. Nucleic Acids

microRNAs are small non-coding single-stranded RNA molecules with a crucial role in the expression of several messenger RNAs (mRNAs) and thus in the regulation of proteins expression. As their abnormal expression is related to cancer, they become important biomarkers for early cancer diagnosis [3,12,26,32,34]. MicroRNA detection is difficult once have small sizes, are present in very low concentrations, and has a high degree of sequence similarity. Conventional methodologies for microRNA analysis are complex and time-consuming, restricting their practical application [3,32]. For that reason, a considerable effort has been made in order to develop highly sensitive and specific methods for microRNA detection [34].

Zheng and co-workers [34] described a lateral-flow nucleic acid biosensor for multiplex detection of microRNA-21, microRNA-155, and microRNA-210, based on sandwich-type nucleic acid hybridization reactions. Biotin-streptavidin-modified single-stranded DNA (ssDNA) probes were immobilized onto cellulose and an AuNP-ssDNA conjugate recognizes the hybridized microRNA. A red coloration, with an intensity proportional to target concentration, was observed indicating the accumulation of AuNPs on test lines. The analytical range of this assay is 0.01 to 5 nM, 0.1 to 10 nM and 0.05 to 10 nM for microRNA-155, microRNA-21 and microRNA-210, respectively. The LOD is 0.073 nM, 0.061 nM and 0.085 nM for microRNA-21, microRNA-155 and microRNA-210, respectively.

Fakhri and co-workers [26] developed a paper-based biosensor for microRNA-21 detection based on peroxidase mimetic activity of DNA-templated Ag/Pt nanoclusters (DNA-Ag/Pt NCs) that catalyze the oxidation of TMB producing a blue color. microRNA-21 can selectively inhibit the peroxidase-like activity of the nanoclusters thus hindering the oxidation of TMB and producing a decrease in blue color intensity with increasing concentrations of target. Image analysis by ImageJ showed that grey intensity values increased with increasing concentrations of microRNA-21. The produced sensor was able to detect microRNA-21 in a linear range from 10 to 1000 pM, with a LOD of 4.1 pM.

Gao and co-workers [32] designed an LFA for microRNA-215 detection based on sandwich-type hybridization reactions among biotin-modified DNA probes, the target, and AuNPs modified with DNA probes. In the presence of the target, AuNPs accumulate on the test line thus leading to visual detection. Qualitative reading was performed by observation of the red band formed on the test line and at least 75 pM of microRNA can be detected, whereas for quantitative measurements the color intensity was captured by a portable strip reader combined with a suitable software being the response linear in a range from 0.075 to 0 nM, with a LOD of 60 pM.

Naorungroj and colleagues [65] developed a smartphone-assisted colorimetric PAD for PoC screening of cervical cancer, based on HPV type 16 DNA detection. Whatman^®^ paper no. 1 was used as substrate and hydrophobic barriers were produced by wax printing, forming a detection/test zone and a control zone. A pyrrolidinyl peptide nucleic acid (acpcPNA) was used as a probe and dextrin-stabilized AuNPs (d-AuNPs) were used as the colorimetric reagent. A complex is formed between the probe and its DNA target and the remaining probe led to different degrees of d-AuNPs aggregation, depending on its amount. A smartphone application, Colorimeter RGB Colorimeter, was used to analyze the color intensity. HPV DNA was linearly detected from 1 to 1000 nM. This sensor showed a high selectivity against single and two-based mismatch and also for non-complementary DNA sequences and was successfully applied to the detection of HPV DNA from cell line samples.

### 6.5. Other Molecules

Apart from the large biomolecules previously discussed (e.g., proteins), small biomolecules, such as extracellular vesicles or metabolites, that result from cellular metabolism, can be used as cancer biomarkers [3]. Citrate concentration in urine relates to kidney disorders and its decrease in the prostatic fluid can be an indicator of prostate cancer at its early stages [42,66].

Abarghoei and colleagues [42] developed a real-time colorimetric paper sensor on a Y-shaped microfluidic device for citrate quantification in urine samples for prostate cancer early detection, by using cysteine-capped gold nanoclusters (Cys-AuNCs) as peroxidase mimetic. In this work, Cys-AuNCs were immobilized onto spot zones, catalyzing the oxidation of TMB in the presence of hydrogen peroxide and thus leading to the production of a bright blue product with an intense absorption peak at 650 nm. When citrate is present in the sample, it binds to Cys-AuNCs and the absorption significantly decreases once it creates a coating around the gold nanocluster, thus inhibiting TMB oxidation. A linear relationship for citrate was observed for the range of 1 μM to 10 mM with a LOD of 0.4 μM.

Shaban and co-workers [66] developed a colorimetric sensor for citrate ions detection on Whatman^®^ filter paper substrate. In this work, AgNPs were capped with two types of surfactants, cetyl trimethyl ammonium bromide (CTAB) and a newly synthesized Gemini (GFEO) surfactant. These dual-surfactant NPs were used for selective and sensitive citrate detection with a limit of detection of 250 µM for naked-eye observation and 4.05 nM using a UV-Vis spectrophotometer. The increase in citrate concentrations originates a color change from yellowish to green due to the aggregation of AgNPs upon citrate addition.

Plasma lysophosphatidic acid (LPA) is a phospholipid with an important role in tumor progression and metastasis. Its levels are high in early-stage ovarian cancer patients, being a more sensitive biomarker than CA-125 for screening early-stage ovarian cancer [33].

Wang and co-workers [33] aimed a colorimetric lateral-flow platform for LPA detection based on polydiacetylenes (PDAs). PDA processes a side-chain that improves the specific recognition of LPA once it interacts with the phosphate head group and the long-chain fatty-acid tail group. In this work, the authors adopted a “lock-key” strategy by using imidazolium functionalized PDAs (iPDAs) to recognize LPA, making use of the synergistic electrostatic and hydrophobic interactions between them. The iPDAs, immobilized on a NC membrane, interact with the LPA present in the samples, which led to a conformation transition in the PDA backbone associated with a blueshift on its absorbance band and consequently, a color change from blue to red that could be observed by naked-eye. This is highly sensitive and enables LPA detection in 5 min.

Exosomes are extracellular vesicles, with 40–200 nm and are present in several biological fluids. These molecules transport several components of the cells as proteins, nucleic acids, and lipids from which they are derived. Tumor-derived exosomes have been studied and can be used as cancer biomarkers [37].

**Figure 10:**
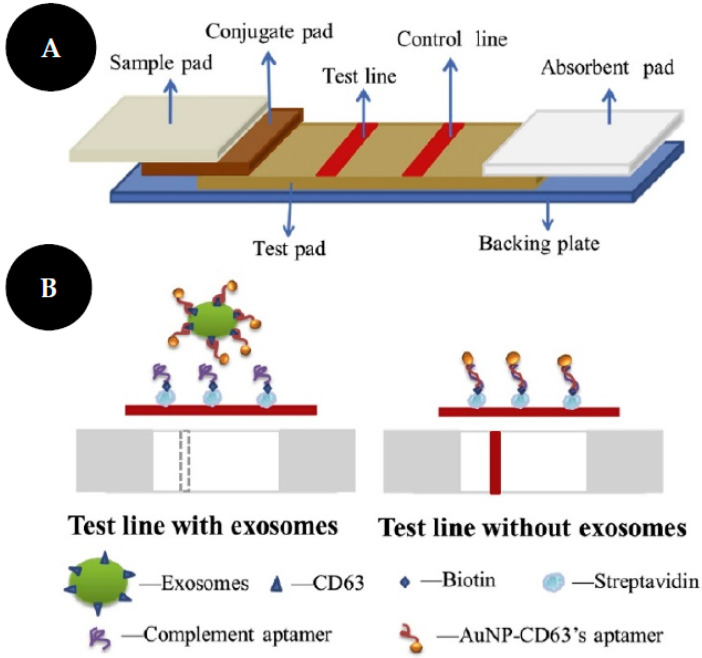
Scheme of LFA test strips (**A**). Scheme of competitive LFA with aptamer as recognition elements for exosome identification (**B**). Reproduced and adapted with permission from [37].

The work of Yu and colleagues [37] consist of an LFIA based on a competitive strategy to detect non-small lung cancer (NSCLC)-derived exosomes (Figure 10). They used an aptamer as a biorecognition element, against CD63 protein on the exosome membrane, due to aptamers’ advantages over Abs, such as high affinity and stability. Thus, a streptavidin-biotin-CD63 aptamer was immobilized on the NC membrane and will compete with the AuNPs functionalized with CD63 aptamer, by a thiol-gold functionalization strategy, that was used as colorimetric probes. In this assay, the amount of exosomes is inversely proportional to the color revealed at the test line. A549 exosomes, isolated from human lung carcinoma cells were identified with an LOD of 6.4 × 10^9^ particles/mL.

## 7. Advantages and Challenges of Cancer Biomarker Detection in Colorimetric PADs

The unique properties of PADs have enabled a low-cost, rapid, and simple test for PoC of several molecules as cancer biomarkers. It uses low sample volumes and often does not require sample pre-treatment. Many of them have long shelf life and do not require refrigeration during storage, being very suitable for developing countries. Additionally, as a naked-eye result is provided, they generally do not require sophisticated equipment for readout [46].

Despite the relevant advantages of using PADs, there are also some disadvantages and challenges [12]. Paper has some physical inherent properties that cannot be rigorously controlled, such as pore size, porosity, surface area, wettability, permeability, and capillary flow rate, that affect the performance of the device regarding fluid flow, color uniformity, and reagents immobilization [5]. For this reason, challenges in the design of new paper-based biosensing devices are directed to the selection of papers with specific properties such as appropriate porosity, paper purity, filtration speed, and pore surface hydrophobicity, among others [12]. Additionally, the retention and evaporation of samples and reagents should be controlled [4]. Physical and chemical treatments are required in order to reduce these batch-to-batch variations [5]. Regarding the fabrication of hydrophobic barriers on μPADs, besides there are several available techniques, they require expensive equipment and skilled personnel [24]. Visual colorimetric detection may also suffer from a lack of reproducibility and sensitivity, leading to the need for the development of more precise and sensitive detection methods [11]. When the target biomarker occurs in high concentrations due to some condition or disease, the sensibility is not a problem, and colorimetric paper-based assays can distinguish the patients from healthy people. However, in some diseases, a specific biomarker is at low concentrations in the early of the disease. In that cases, a highly sensitive assay is required [5,13]. Limited sensitivity can be surpassed with alternative materials that enable a better recognition of the target and a separation of the interfering substances [5]. Sensitivity enhancement approaches could also be used in the detection of trace levels biomarkers [3]. For example, silver and enzymes have been used to amplify the signal of AuNPs [46].

In addition, many PADs allow only qualitative or semi-quantitative results, which is a major drawback of these devices, that can be overlapped by the development of new readout techniques [3]. Some electrochemical or optical techniques, (e.g., fluorescence) can be used to improve LODs on colorimetric PADs and enable quantitative measurements. However, we need to be aware that they imply higher costs and complexity [11,36]. Luminescent labels have been used for fluorescent, chemiluminescent, or electro-chemiluminescent detection. These types of signals, such as LOD, are lower than those used in colorimetric detection. However, they require specific readers, increasing the cost and complexity of the assay [5]. Additionally, the naked-eye observation of a colorimetric assay response could lead to subjective results. For this reason, for a quantitative colorimetric assay, a clear color change should occur, sometimes using signal amplification strategies and an adequate tool should be used to evaluate the signal by digitalizing the color and providing numeral data [5]. However, external factors such as light conditions while capturing results can influence their quality. Another limitation of colorimetric PADs is their less appropriateness for impaired and color-blind people [17].

Selectivity is also an important factor as complex samples are often used in these types of sensors and components in the matrix should not interfere with the analysis [11]. Since the diagnosis of a disease generally requires the measurement of multiple parameters, the ability of the device to simultaneously detect several parameters, also called multiplexing, should be considered, especially in a complex disease such as cancer [11]. To this end new, detection techniques and different designs such as strip arrays, star shapes, or multiple parallel channels have been applied. However, it should be noted that multiplex assays could suffer from a lack of specificity due to cross-reaction, especially with complex samples [3,5].

Stability is a critical factor in the feasibility of the sensor and an ideal device should be designed to be a long shelf-life maintaining its stability under inappropriate storage conditions [5,11]. Temperature, pH, and humidity are environmental factors that could impact the quality of the PAD [5]. The preservation of the activity of the biological molecules (e.g., Abs, enzymes, redox probes) stored in cellulose pores is a critical deal for these types of sensors. For this reason, some natural materials have been replaced by alternative materials with high stability as aptamers or other artificial receptors [5].

Developments of PADs are gaining great attention and are coupled with smartphones for application in clinical diagnosis as they provide real-time and on-site measurements. Even though there have been a number of PoC systems developed in the last decade, most of them have either not been commercialized or widely established in the market due to the lack of integration and automation of the designs [18,56]. Despite LFAs and dip-sticks having been successfully commercialized, there are only a few examples of μPADs in the market [11]. It could be understood that these types of devices are in growing development and will become more available as complementary or alternative tools for biomarker detection, thus simplifying diagnosis and prognosis of a wide range of analytes and consequently improving life quality [4].

## 8. Conclusions and Future Perspectives

In clinical practice, a cancer diagnosis is performed by complex methods that, although highly sensitive and specific, are not suitable for routine diagnosis. Since paper is a versatile substrate with inherent properties and the possibility of several modifications, PADs represent a new approach that provides a suitable alternative to conventional methods and meets the requirements of a PoC device. Colorimetric PADs have been used for the detection of several analytes, as cancer biomarkers in different types of samples.

The current review elucidates the application of paper as a substrate in sensor devices and the use of CM for signal transduction and detection of cancer biomarkers.

The major advances in PADs development over the last years have been made possible by improvements in signal-amplification strategies. This has been made possible by the use of novel materials, such as new NPs that have been used as labels to increase sensitivity and provide a clear signal based on color change. The combination of PADs and technologies, such as smartphones should move toward better qualitative and quantitative signals allowing a preliminary on-site disease screening outside the laboratory or the hospital, as in remote locations.

Despite the considerable number of reports on the use of PADs for colorimetric detection of biomarkers, such as cancer, the real-world applications are still very limited. Notwithstanding, the current development in this field, more research is needed to identify and address several pitfalls of paper-based biosensors to understand their drawbacks and develop strategies to overcome them, thereby improving their performance and enabling their commercialization. These challenges include low analytical performance, such as high limits of detection, insufficient specificity, poor stability, the need for multiplexing, and subjective interpretation of the results.

## Figures and Tables

**Table 1:** Colorimetric paper-based sensors for cancer biomarkers reported in the last 12 years.

Target		Type of PAD	Recognition Element	System of Detection	Linear Range	LOD	Reference
Proteins	CEA	μPAD	Ab	HRP + TMB	0.5 to 70 ng/mL	0.015 ng/mL	[1]
		μPAD	Ab	HRP + TMB	0 to 40 ng/mL	2 ng/mL	[2]
		spot test	Ab	nanocomposite with peroxidase-like activity + TMB	0.002 to 75 ng/mL	0.51 pg/mL	[3]
		spot test	Ab	Carbon nanotubes with peroxidase-like activity + TMB	0.005 to 30 ng/mL	2.6 pg/mL	[4]
		spot test	Ab	Biotin + avidin-peroxidase + TMB	-	-	[5]
		μPAD	Ab	HRP + TMB	0.1 to 20.0 ng/mL	0.03 ng/mL	[6]
		μPAD	Ab	AuNPs + Pd/Fe_3_O_4_@C NPs with peroxidase-like activity + TMB	0.005 and 30 ng/mL	1.7 pg/mL	[7]
	CA-125	spot test	Ab	AuNPs + silver enhancement	30 to 100 U/mL.	30 U/mL	[8]
		spot test	Ab	Cys-AuNPs	0.1 to 10 ng/mL	0.413 ng/mL	[9]
	α-AFP	spot test	Ab	Cys-AuNPs	0.1 to 100 ng/mL	1.054 ng/mL	[9]
		μPAD	Ab	AuNPs + Pd/Fe_3_O_4_@C NPs with peroxidase-like activity + OPD	0.005 and 30 ng/mL	1.7 pg/mL	[7]
		spot test	Ab	AuNPs	10 to 400 ng/mL	0.17 pg/mL	[10]
	PSA	μPAD	Ab	AuNPs	0.5 to 50 μg/L	≈360.2 ng/L	[11]
		μPAD	Ab	Fe_3_O_4_ conversion to PB	3 to 80 ng/mL	2.7 ng/mL	[12]
	PCA3	μPAD	-	chromogenic substrate	1 × 10^−3^ to 1 × 10^1^ pg/μL	0.34 fg/μL	[13]
	p16^INK4a^	spot test	Ab	AuNPs peroxidase-like activity + HRP + TMB	-	300 cells (HeLa and CasKi cell lines) and 3000 cells (SiHa cell lines)	[14]
	HPV 16/18 E6	spot test	Ab	AuNPs peroxidase-like activity + HRP + TMB	-	0.0005 pg/mL	[15]
	VCP	LFA	Ab	gold-coated magnetic nanostructures + streptavidin poly-HRP + TMB	-	25 fg/mL	[16]
	sarcosine	spot test	sarcosine oxidase	sarcosine oxidase + HRP + ABTS	0 to 1 mM	0.21 mM	[17]
		spot test	sarcosine oxidase	sarcosine oxidase + HRP + TMB	0 to 10 μM	0.6 μM	[18]
	Cyt c	µPAD	aptamer	mesoporous carbon + TMB	1 µM to 1 mM	5.0 × 10^−7^ M	[19]
	IL-6	LFA	Ab	AuNPs	1.25 to 9000 ng/mL	0.38 ng/mL	[20]
Enzymes	telomerase	spot test	telomere complementary oligonucleotide	AuNPs + telomerase elongated biotinylated probe	6 to 25,000 cells	6 cells	[21]
		spot test	telomerase substrate oligonucleotide	MB	0 to 100 cells/μL	20 cells/μL	[22]
	ALP	µPAD	-	PNPP	30–500 U/mL	-	[23]
	PEAK1	spot test	Ab	AuNPs + HNB	1 × 10^−9^ g/mL to 1 × 10^−5^ g/mL	1 ng/mL	[24]
Growth factors	HER2	LFA	aptamer	AuNPs + biotin + streptavidin	0 to 50 nM	20 nM	[25]
Nucleic acids	microRNA-21	LFA	ssDNA	AuNPs + biotin + streptavidin	0.1 to 10 nM	0.073 nM	[26]
		µPAD	ssDNA	Ag/Pt NCs with peroxidase-like activity + TMB	10 to 1000 pM	4.1 pM	[27]
	microRNA-155	LFA	ssDNA	AuNPs + biotin + streptavidin	is 0.01 to 5 nM	0.061 nM	[26]
	microRNA-210	LFA	ssDNA	AuNPs + biotin + streptavidin	0.05 to 10 nM	0.085 nM	[26]
	microRNA-215	LFA	ssDNA	AuNPs + biotin	0.075 to 0 nM	60 pM	[28]
	HPV type 16 DNA detection	spot test	acpcPNA	AuNPs	1 to 1000 nM	1 nM	[29]
Other molecules	citrate	µPAD	-	Cys-AuNCs with peroxidase-like activity + TMB	1 μM to 10 mM	0.4 μM	[30]
		spot test	-	AgNPs	100 to 1000 μM	4.05 nM	[31]
	LPA	LFA	PDA	conformation transition	-	-	[32]
	NSCLC-derived exosomes	LFA	aptamer	AuNPs + biotin + streptavidin	-	6.4 × 10^9^ particles/mL	[33]

(Ab: antibody; ABTS: 2,2-azino-bis(3-ethylbenzothiazoline-6-sulfonic acid) diammonium salt; acpcPNA: pyrrolidinyl peptide nucleic acid; ALP: alkaline phosphatase; AuNPs: gold nanoparticles; CA-125: cancer antigen 125; CEA: carcinoembryonic antigen; Cys: cysteine; Cyt c: cytochrome c; HER2: epidermal growth factor receptor 2; HNB: hydroxyl naphthol blue; HPV: human papilloma virus; HRP: horseradish peroxidase; IL-6: interleukin-6; LFA: lateral-flow assay; LPA: plasma lysophosphatidic acid; MB: methylene blue; NC: nanoclusters; NPs: nanoparticles; NSCLC: non-small lung cancer; OPD: orto-phenylenediamine; PB: prussian blue; PCA3: prostate cancer antigen 3; PDA: polydiacetylene; PEAK1: pseudopodium-enriched atypical kinase one; PNPP: para-nitrophenylphosphate; PSA: prostate-specific antigen; ssDNA: single stranded DNA; TMB: tetramethylbenzidine; VCP: valosin-containing protein; α-AFP: alpha-fetoprotein; μPAD: microfluidic paper-based device).

## Data Availability

Not applicable.
